# The prognostic significance of synchronous metastasis in glioblastoma multiforme patients: a propensity score-matched analysis using SEER data

**DOI:** 10.3389/fneur.2024.1429826

**Published:** 2024-10-08

**Authors:** Hui Shen, Qing Mei, Xubin Chai, Yuanfeng Jiang, Aihua Liu, Jiachun Liu

**Affiliations:** ^1^Department of Interventional Neuroradiology, Sanbo Brain Hospital, Capital Medical University, Beijing, China; ^2^Department of Interventional Neuroradiology, Beijing Tiantan Hospital, Beijing Neurosurgical Institute, Capital Medical University, Beijing, China; ^3^Department of Neurology, Beijing Pinggu Hospital, Beijing, China; ^4^State Key Laboratory of Brain and Cognitive Science, Institute of Biophysics, Chinese Academy of Sciences, Beijing, China; ^5^Department of Neurosurgery, Beijing Hospital, Beijing, China

**Keywords:** glioblastoma multiforme, synchronous metastasis, propensity score matching, cancer-specific survival, SEER

## Abstract

**Background:**

Glioblastoma multiforme (GBM) with synchronous metastasis(SM) is a rare occurrence. We extracted the data of GBM patients from the SEER database to look into the incidence of SM in GBM, determine the prognostic significance of SM in GBM, and assess therapeutic options for patients presenting with SM.

**Methods:**

From 2004 to 2015, information on GBM patients was obtained from the Surveillance, Epidemiology, and End Results (SEER) database. The propensity score matching (PSM) method was employed to mitigate confounding factors between SM and non-SM groups, subsequently investigating the prognostic significance of SM in patients with GBM. Multivariate Cox proportional hazards regression analyses were employed to identify independent prognostic variables for GBM patients with SM. A forest plot was used to visualize the results.

**Results:**

A cohort of 19,708 patients was obtained from the database, among which 272 (1.4%) had SM at the time of diagnosis. Following PSM at a 3:1 ratio, in both univariate and multivariate cox regression analysis, SM (HR = 1.27, 95% CI: 1.09–1.46) was found to be an independent predictive predictor for GBM patients. Furthermore, the Cox proportional hazard forest plot demonstrated that independent risk variables for GBM patients with SM included age (Old vs. Young, HR = 1.44, 95% CI: 1.11–1.88), surgery (biopsy vs. no surgery, HR = 0.67, 95% CI: 0.46–0.96;Subtotal resection vs. no surgery, HR = 0.47, 95% CI: 0.32–0.68;Gross total resection vs. no surgery, HR = 0.44, 95% CI: 0.31–0.62), radiotherapy (HR = 0.58, 95% CI: 0.41–0.83), and chemotherapy (HR = 0.51, 95% CI: 0.36–0.72).

**Conclusion:**

The predictive value of SM in GBM was determined by this propensity-matched analysis using data from the SEER database. Radiotherapy, chemotherapy, and surgery constitute an effective treatment regimen for patients with SM. A more positive approach toward the use of aggressive treatment for GBM patients with SM may be warranted.

## Introduction

Glioblastoma multiforme (GBM) is the most common type of primary malignant brain tumor in adults, distinguished by a dismal prognosis and poor quality of life. Intracranial and extracranial metastases have been documented in 1–2% (1) and 0.4–0.5% (2, 3) of cases, occurring late in the course of the disease, respectively. The prevalence rates might be underestimated since metastatic screening is not a standard practice. Synchronous metastasis(SM) of GBM is even more uncommon, with only a few case reports in the literature (4). The precise incidence of SM in GBM remains elusive.

Metastasis holds critical importance for the prognosis of GBM, as evidenced by a comprehensive literature review (5). Several studies have shown the intensive treatment can prolong survival time for certain individuals with metastases (6–8). However, these researches are confined to tiny sample size, and susceptible to selection bias. Therefore, it is essential to uncover additional information regarding prognostic factors and treatment strategies for these patients.

The present study aimed to use large, population-based cancer registry data to investigate both the incidence and risk factors of SM in patients with GBM. Additionally, it seeks to identify prognostic factors and formulate treatment strategies for patients with SM.

## Patients and methods

### Patients

The patient cases were enrolled in the study based on the latest version of the publicly available SEER17 database (published in November 2022) by using SEER*Stat 8.4.2.^1^

**Figure 1:**
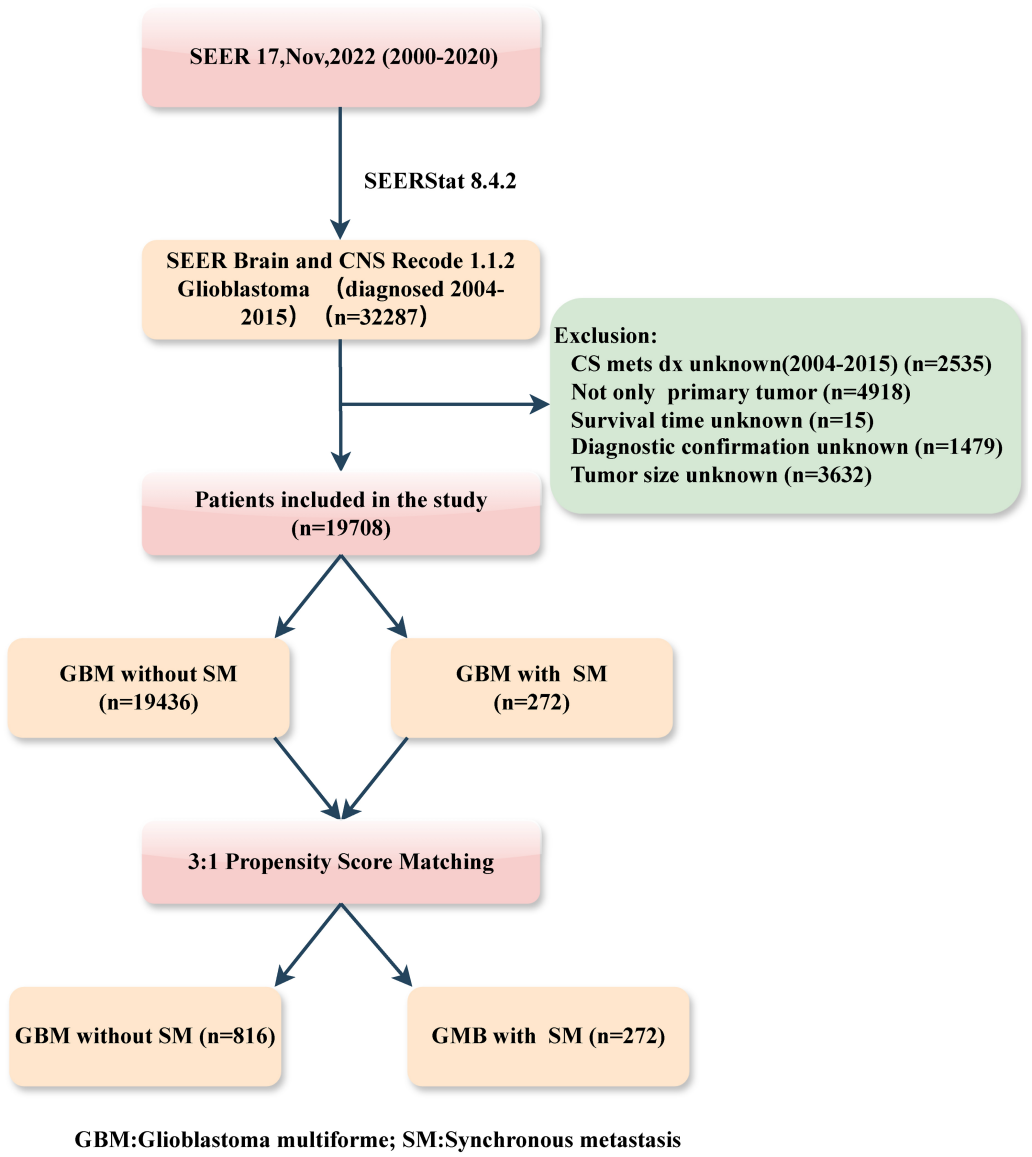
Flowchart illustrating patient selection of this study.

Patients diagnosed with primary GBM, both with and without SM, between the years 2004 and 2015, were retrospectively identified in this study. The inclusion criteria were as follows: (1) histology diagnosis with GBM (SEER Brain and CNS Recode 1.1.2 GB); (2) year of diagnosis (range: 2004–2015). While the exclusion criteria included: (1) information on SM (CS mets dx) unknown; (2) not only primary tumor; (3) patient with unknown survival time; (4) incomplete or unconfirmed diagnoses, and (5) tumor size unknown. The patient selection flowchart from the SEER database is delineated in Figure 1.

The following variables were incorporated in the current study: demographic characteristics (year, age, sex, race, marital status, household income, rural–urban area); clinicopathological information (primary site, laterality, tumor size, SM, surgery, radiotherapy and chemotherapy), and follow-up data (cause-specific death and survival time).

Age at diagnosis as a continuous variable was separated into young group (<60 years) and old group (≥60 years). Median household income inflation-adjusted to 2021 was used to classify individuals into the low-income ($60,000 or less) and high-income ($60,001 or more) groups. Tumor size was characterized by the dimensions of the primary tumor. The surgical interventions, which pertain to the resection of the primary tumor, were systematically documented using a specific coding schema.: gross total resection (GTR) was designated with codes 30 or 55; subtotal resection (STR) was represented by codes 22, 21, or 40; biopsy was assigned codes 10, 20, or 90; and instances where no surgery was performed were denoted with code 0. SM: Defined as intracranial and/or extracranial metastatic lesions resulting from the spread of a primary GBM at the initial diagnosis. In the SEER database, SM, which includes both extracranial and intracranial metastasis, is denoted by the term “CS mets dx.” Extracranial metastasis is denoted by codes 30 or 50, whereas intracranial metastasis by codes 10 or 20. Cancer-specific survival (CSS) is defined as the duration between the primary diagnosis and the date of death associated with GBM.

### Statistical analysis

A Propensity Score Matching (PSM) analysis was utilized to adjust for all other variables between patients with and without SM. The “Matchit” package in R was employed to match the propensity scores between the two groups, employing the nearest neighbor algorithm with a matching ratio of 1:3. Then, Pearson’s Chi-squared test (“gtsummary” package) was used to compare baseline characteristics between groups. Categorical variables are presented as proportions and percentages of the total. Cox proportional hazards regression analysis was performed to evaluate the prognostic effect of SM in GBM (“survival” package). The effect of these confounders was quantified by calculating the changes in the effect size of SM for GBM when each variable is added to the model sequentially in a step-wise fashion (“chest” package) (9). Based on the observed effects, a multivariable Cox proportional hazards regression analysis was conducted. Furthermore, the Kaplan–Meier method and log-rank analysis were utilized to depict the CSS curves before and after PSM in both groups (“survminer” package).

Cox proportional hazards regression models, both univariable and multivariable, were used to identify the prognostic factors for GBM patients with SM. Moreover, a forest plot (“forestmodel” package) was constructed to visualize the findings.

In our study, statistical significance was defined as a two-tailed *p* value<0.05. The statistical analysis was performed with R program (version 4.3.2).

## Results

### Baseline characteristics before and after PSM

Based on inclusion and exclusion criteria, a total of 19,708 GBM patients were selected from the SEER database. Demographical and clinical characteristics for GBM with or without SM were shown in Table 1. Patients diagnosed with SM tended to be non-white, infratentorial, and not to have a paired site (*p* < 0.05); additionally, they were less likely to have undergone radiotherapy, chemotherapy, or surgery (*p* < 0.001).

**Table 1:** Characteristics of glioblastoma multiforme patients before and after propensity score matching.

Characteristic	Before matching			After matching^3^		
	Non-SM	SM		Non-SM	SM	
	*N* = 19,436^1^	*N* = 272	*p*-value^2^	*N* = 816	*N* = 272	*p*-value^2^
Year, *n* (%)			0.10			0.7
2004–2007	5,559 (29%)	71 (26%)		205 (25%)	71 (26%)	
2008–2011	6,453 (33%)	80 (29%)		262 (32%)	80 (29%)	
2012–2015	7,424 (38%)	121 (44%)		349 (43%)	121 (44%)	
Age, *n* (%)			0.5			0.8
Young	8,386 (43%)	112 (41%)		344 (42%)	112 (41%)	
Old	11,050 (57%)	160 (59%)		472 (58%)	160 (59%)	
Sex, *n* (%)			0.3			0.4
Female	8,022 (41%)	120 (44%)		338 (41%)	120 (44%)	
Male	11,414 (59%)	152 (56%)		478 (59%)	152 (56%)	
Race, *n* (%)			<0.001			0.6
Others	2,049 (11%)	47 (17%)		129 (16%)	47 (17%)	
White	17,387 (89%)	225 (83%)		687 (84%)	225 (83%)	
Marital status, *n* (%)			0.6			0.4
Divorced	6,964 (36%)	102 (38%)		285 (35%)	102 (38%)	
Married	12,472 (64%)	170 (63%)		531 (65%)	170 (63%)	
Household income, *n* (%)			0.8			0.3
<60,000	5,766 (30%)	79 (29%)		209 (26%)	79 (29%)	
60,000+	13,670 (70%)	193 (71%)		607 (74%)	193 (71%)	
Rural urban, *n* (%)			0.6			0.8
Metropolitan	17,017 (88%)	241 (89%)		728 (89%)	241 (89%)	
Nonmetropolitan	2,419 (12%)	31 (11%)		88 (11%)	31 (11%)	
Tumor size, *n* (%)			0.4			0.8
<4.5 cm	9,443 (49%)	139 (51%)		409 (50%)	139 (51%)	
4.5 cm+	9,993 (51%)	133 (49%)		407 (50%)	133 (49%)	
Primary site, *n* (%)			<0.001			0.7
Frontal lobe	5,574 (29%)	79 (29%)		211 (26%)	79 (29%)	
Parietal lobe	3,160 (16%)	52 (19%)		172 (21%)	52 (19%)	
Temporal lobe	5,007 (26%)	41 (15%)		136 (17%)	41 (15%)	
Others	5,695 (29%)	100 (37%)		297 (36%)	100 (37%)	
Laterality, *n* (%)			0.010			0.9
Left	8,376 (43%)	113 (42%)		336 (41%)	113 (42%)	
Not a paired site	2,580 (13%)	53 (19%)		171 (21%)	53 (19%)	
Right	8,480 (44%)	106 (39%)		309 (38%)	106 (39%)	
Surgery, *n* (%)			<0.001			0.7
NS	3,240 (17%)	81 (30%)		262 (32%)	81 (30%)	
Biopsy	3,657 (19%)	56 (21%)		152 (19%)	56 (21%)	
STR	3,015 (16%)	57 (21%)		188 (23%)	57 (21%)	
GTR	9,524 (49%)	78 (29%)		214 (26%)	78 (29%)	
Radiotherapy, *n* (%)	15,128 (78%)	179 (66%)	<0.001	553 (68%)	179 (66%)	0.6
Chemotherapy, *n* (%)	13,822 (71%)	152 (56%)	<0.001	481 (59%)	152 (56%)	0.4

^1^*n* (%), ^2^Pearson’s Chi-squared test; ^3^Nearest Neighbor Matching with a ratio 1:3. NS, No surgery; STR, Subtotal resection; GTR, Gross total resection; SM, Synchronous metastasis.

After 1:3 PSM, 1088 patients were matched, which included 272 patients with SM and 816 patients without SM. All demographic and clinical characteristics were well-matched, suggesting that the PSM effectively minimized potential selection bias (*p* > 0.05).

### The prognostic effect of SM in patients after PSM

**Figure 2:**
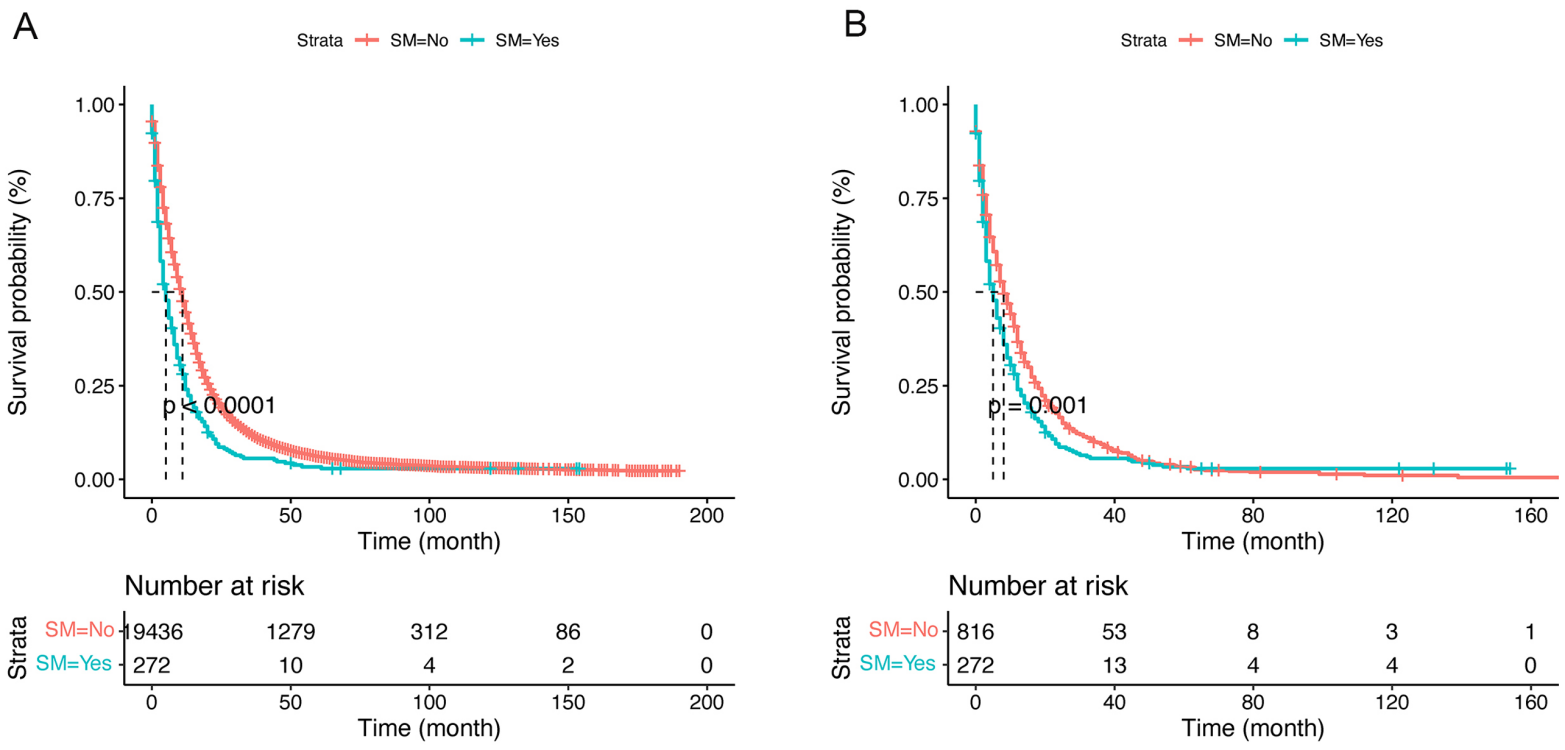
Kaplan–Meier curves of cause-specific survival (CSS) by synchronous metastasis (SM) before **(A)** and after **(B)** propensity score matching (PSM).

The univariate Cox proportional hazards model demonstrated a significant increment in mortality risk for GBM patients with SM after PSM (HR: 1.27, 95% CI:1.10–1.46, *p* < 0.00; Table 2). Moreover, Kaplan–Meier analysis curves comparing CSS for SM and non-SM groups before and after PSM are depicted in Figure 2.

**Table 2:** Univariate and multivariate analyses of cancer-specific survival (CSS) in the cohort after propensity score matching.

Characteristic	Univariate			Multivariate		
	HR^1^	95% CI^1^	*p*- value	HR^1^	95% CI^1^	*p*- value
Year						
2004–2007	—	—				
2008–2011	0.81	0.69, 0.96	0.012			
2012–2015	0.80	0.69, 0.93	0.005			
Age						
Young	—	—		—	—	
Old	1.93	1.70, 2.18	<0.001	1.77	1.54, 2.03	<0.001
Sex						
Female	—	—				
Male	0.94	0.83, 1.06	0.33			
Race						
Others	—	—				
White	1.06	0.89, 1.25	0.53			
Marital status						
Divorced	—	—				
Married	1.00	0.88, 1.13	>0.99			
Household income						
<60,000	—	—				
60,000+	1.01	0.88, 1.16	0.91			
Rural urban						
Metropolitan	—	—				
Nonmetropolitan	1.09	0.90, 1.32	0.40			
Primary site						
Frontal lobe	—	—		—	—	
Parietal lobe	0.95	0.80, 1.14	0.60	0.90	0.75, 1.08	0.3
Temporal lobe	1.02	0.84, 1.23	0.88	1.00	0.82, 1.22	>0.9
Others	1.22	1.04, 1.42	0.012	1.16	0.99, 1.37	0.067
Laterality						
Left	—	—				
Not a paired site	1.29	1.10, 1.52	0.002			
Right	1.01	0.88, 1.16	0.85			
Tumor size(cm)						
<4.5 cm	—	—		—	—	
4.5 cm+	1.07	0.95, 1.21	0.27	1.15	1.02, 1.31	0.028
SM						
No	—	—		—	—	
Yes	1.27	1.10, 1.46	<0.001	1.27	1.09, 1.46	0.001
Surgery						
NS	—	—		—	—	
Biopsy	0.57	0.48, 0.68	<0.001	0.61	0.50, 0.73	<0.001
STR	0.47	0.39, 0.55	<0.001	0.54	0.45, 0.65	<0.001
GTR	0.42	0.35, 0.49	<0.001	0.43	0.36, 0.51	<0.001
Radiotherapy						
No	—	—		—	—	
Yes	0.41	0.36, 0.47	<0.001	0.58	0.48, 0.71	<0.001
Chemotherapy						
No	—	—		—	—	
Yes	0.43	0.38, 0.48	<0.001	0.59	0.49, 0.71	<0.001

^1HR, Hazard Ratio; CI, Confidence Interval; NS, No surgery; SM, Synchronous metastasis; STR; Subtotal resection; GTR, Gross total resection.^

**Figure 3:**
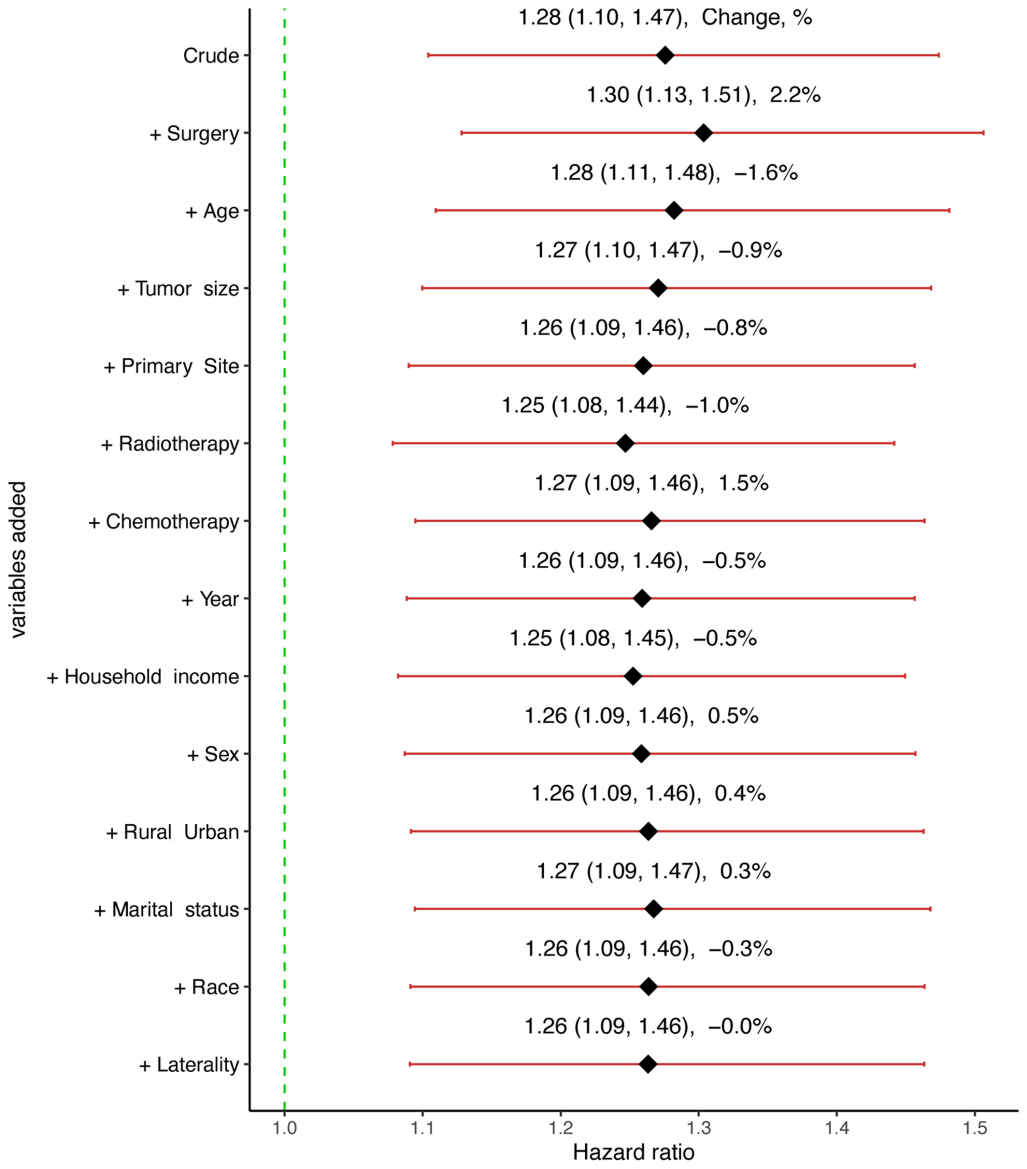
Graphical representation of the change in the estimate of the effect of synchronous metastasis (SM) on cause-specific survival (CSS) with each variable added to the multivariable Cox regression model.

The confounding effect of each remaining variable on the association of SM on the CSS of GBM patients was quantified and is presented in Figure 3. The results remain stable with each variable being added to multivariable Cox proportional hazards model sequentially in a step-wise fashion. Based on the effects and clinical experience, multivariable Cox proportional hazards model was established. After adjusting for potential confounding variables including age, primary site, tumor size, surgery, chemotherapy and radiotherapy, SM was still found to be an independent risk factor (HR:1.28, 95% CI:1.11–1.48, *p* < 0.001) in GBM patients (Table 2).

### Prognostic factors for GBM patients with SM

**Figure 4:**
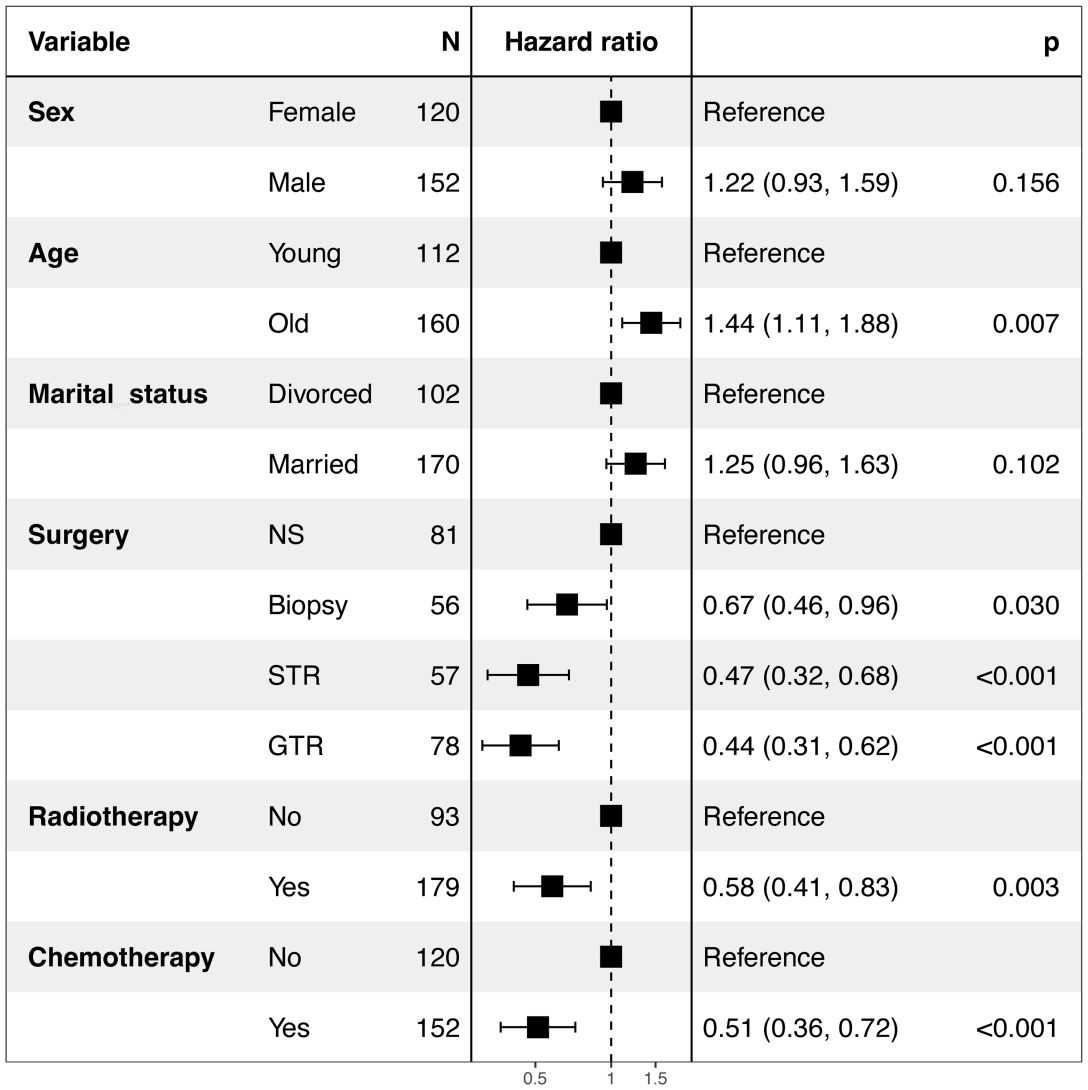
Forest plot of the multivariate Cox analysis of CSS in GBM patients with SM. NS, No surgery; STR, Subtotal resection; GTR, Gross total resection; SM, Synchronous metastasis.

**Figure 5:**
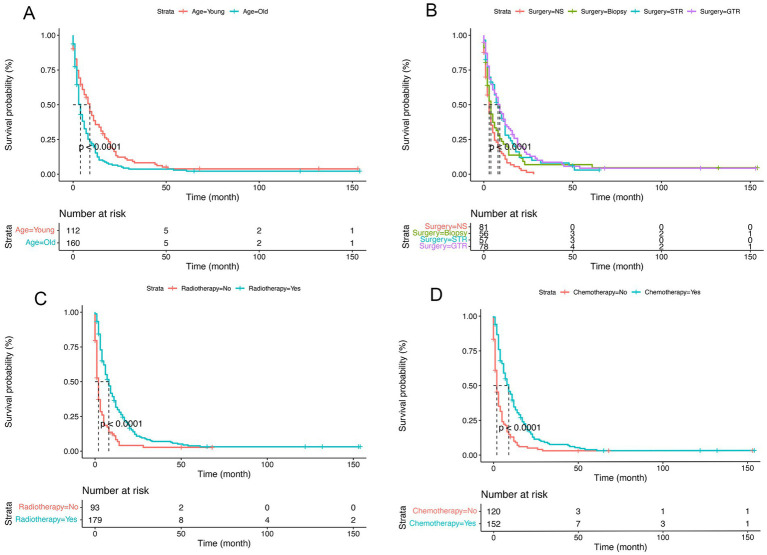
Kaplan–Meier survival curves comparing the cancer-specific survival (CSS) of glioblastoma multiforme (GBM) patients with synchronous metastasis (SM). **(A)**, Age; **(B)**, Surgery; **(C)**, Radiotherapy; **(D)**, Chemotherapy; NS, No surgery; STR, Subtotal resection; GTR, Gross total resection.

In the present research, a cohort of 272 patients diagnosed with GBM exhibiting SM were analyzed to identify and evaluate potential prognostic factors. As delineated in Table 1, the majority were male (56%), white (83%), and diagnosed between 2012 and 2015 (44%). And a total of 191 (70%) patients underwent surgical intervention, 179 (66%) received radiotherapy, and 152 (56%) were administered chemotherapy. Prognostic factors were identified using multivariate Cox regression analyses, revealing that for GBM patients with SM, age (*p* = 0.007), surgery (*p* < 0.001), chemotherapy (*p* < 0.001), and radiotherapy (*p* = 0.003) were independent prognostic factors (Figure 4). The Kaplan–Meier curves of the age, surgery, radiotherapy and chemotherapy subgroups are displayed in Figure 5.

### The prognostic effect of different types of SM in patients

Of these, 272 (1.4%) had SM, with 29 (0.1%) presenting extracranial metastasis and 243 (1.3%) presenting intracranial metastasis. Compared to those with extracranial metastasis, patients with intracranial metastasis were more likely to undergo GTR (30% vs. 14%) (Supplementary Table S1). The Kaplan–Meier curve and forest plot generated using the chest package (Supplementary Figure S1) revealed no statistically significant differences between the two groups.

## Discussion

GBM represents the most prevalent form of malignant primary brain tumors in adults (10), with a 5-year overall relative survival of 6.9% (11). GBM patient with SM is uncommon (12), and the prognostic factors and optimal therapeutic approaches for these patients have not yet been fully elucidated. In this population-based study, we employed the SEER database to conduct a comprehensive analysis of patients diagnosed with GBM, both with and without SM, between the years 2004 and 2015. SM is extremely rare, affecting 1.4% of all patients with GBM in our cohort. We found that SM was an independent prognostic factor for CSS in patients with GBM before and after PSM. Additionally, the analysis also revealed age, surgery, chemotherapy, and radiation therapy as significant prognostic indicators for patients with SM. Notably, the statistical analysis did not discern a significant difference between intracranial and extracranial metastasis, potentially due to the constrained sample size of patients with SM.

Previous studies (12–14) have demonstrated SM is a significant prognostic factor for GBM, yet the exact value of this prognosis remains unknown. Amelot et al. (15) conducted a retrospective analysis of GBM patients with spinal cord metastasis in a French database between January 2004 and 2016, accompanied by a review of the pertinent literature. They found that spinal cord metastasis is associated with a poor prognosis. In our study, to balance baseline confounding factors, we employed PSM analysis to ascertain the exact value of SM for patients with GBM. After adjusting for various covariates, SM consistently emerged as an independent risk factor in GBM patients. This finding underscores the importance of conducting a comprehensive assessment of synchronous metastasis status at the time of initial diagnosis.

The underlying mechanism accounting for SM remains elusive (16). The majority of GBM metastases occur within the central nervous system, which is believed to be attributed to white matter tract infiltration and cerebrospinal fluid seeding (17–19). Extracranial metastases are documented in approximately 0.4–0.5% of all patients (2, 3, 20), suggesting that such interventions may facilitate the dissemination of tumor cells beyond the central nervous system. The presence of extracranial metastasis may be indicative of the existence of circulating tumor cells (CTCs) within the bloodstream. These cells exhibit advanced characteristics, such as epithelial-to-mesenchymal transition and dormancy (21, 22), which are crucial to survive in the bloodstream. And the detection of glioblastoma CTCs holds significant clinical promise for the early diagnosis and prognosis (23, 24).

The standard treatment of primary GBM is maximal safe resection followed by concomitant radiotherapy and temozolomide chemotherapy and then adjuvant temozolomide (25). However, due to the scarcity of case reports, the optimal treatment strategy for patients with SM has yet to be fully established. A meta-analysis of individual patient data, encompassing 115 younger patients from 1928 to 2013, indicated that while a survival benefit could not be statistically validated, an aggressive treatment approach may be ethically justified whenever feasible (6). Furthermore, another meta-analysis revealed that surgical intervention, chemotherapy, and radiotherapy significantly prolongs survival in GBM patients with metastasis (7). In our study, we discovered that surgical intervention exerted a significant impact on the management of patients with SM. Gross total resection, subtotal resection, and biopsy can significantly enhance the cancer-specific survival of patients compared to those no surgical intervention. This indicated that surgical resection can effectively decrease the tumor mass, consequently restoring neurological functions. Additionally, we identified both radiation therapy and chemotherapy as significant prognostic factors. Consistent with a previous study, patients who underwent an aggressive treatment, including surgery, radiation, chemotherapy, and cerebrospinal fluid shunting, demonstrated favorable prognosis (26). Overall, our results suggested that a combination of surgical intervention, chemotherapy, and radiation constitutes an effective therapeutic strategy for patients with SM. Therefore, GBM patients with SM may experience enhanced benefits from more aggressive therapeutic regimens. Nonetheless, additional studies are required to validate these preliminary findings.

Several limitations of our study need to be recognized. Firstly, in the retrospective study, the restricted sample size of patients with SM (*N* = 272) might have a risk of selection bias. Therefore, prospective registry studies with larger sample sizes are necessary to validate our findings. Secondly, as our study included patients from 2004 to 2015 and utilized diagnostic criteria established prior to 2016, these were solely based on pathological diagnosis without integrating molecular diagnosis. Thirdly, information was gathered when the diagnosis was initially made from the SEER database, excluding any metastasis that developed subsequently. Fourthly, the SEER database lacks comprehensive details regarding specific chemotherapeutic agents used, including dosages, treatment duration, and patient responses. Details pertaining to radiation therapy and surgical procedures for metastasis are also missing. These limitations may hinder a thorough evaluation of the efficacy of various treatment modalities.

## Conclusion

In conclusion, the propensity-matched analysis has identified the prognostic value of SM in patients with GBM based on the SEER database. Our findings suggest that surgery, chemotherapy, and radiotherapy constitute an effective therapeutic strategy for GBM patients with SM. However, further research is necessary to confirm these findings through prospective registry studies with larger sample size.

## Data Availability

The original contributions presented in the study are included in the article/Supplementary material, further inquiries can be directed to the corresponding authors.

## Footnotes

^1^https://seer.cancer.gov/
