# Supplementary material for: The prognostic significance of synchronous metastasis in glioblastoma multiforme patients: a propensity score-matched analysis using SEER data
Source: Front Neurol. 2024 Oct 8;15:1429826. doi: 10.3389/fneur.2024.1429826 (PMC11493671; doi:10.3389/fneur.2024.1429826)
Supplement: Supplementary file 1 [file Data_Sheet_1.docx]

Supplementary figure 1

Kaplan–Meier survival curve (A) comparing the cancer-specific survival of glioblastoma multiforme patients with different types of SM and graphical representation (B) of the change in the estimate of the effect of different types of SM on cause-specific survival with each variable added to the multivariable Cox regression model.
