# Supplementary material for: The prognostic significance of synchronous metastasis in glioblastoma multiforme patients: a propensity score-matched analysis using SEER data
Source: Front Neurol. 2024 Oct 8;15:1429826. doi: 10.3389/fneur.2024.1429826 (PMC11493671; doi:10.3389/fneur.2024.1429826)
Supplement: Supplementary file 2 [file Table_1.docx]

**Supplementary table 1** Characteristics of glioblastoma multiforme patients with SM

| **Characteristic** | **Overall**  N = 272^1^ | **SM** | | **p-value**^2^ |
| --- | --- | --- | --- | --- |
|  |  | **Extracranial**  N = 29 | **Intracranial**  N = 243 |  |
| **Age, n (%)** |  |  |  | 0.4 |
| Young | 112 (41%) | 10 (34%) | 102 (42%) |  |
| Old | 160 (59%) | 19 (66%) | 141 (58%) |  |
| **Sex, n (%)** |  |  |  | >0.9 |
| Female | 120 (44%) | 13 (45%) | 107 (44%) |  |
| Male | 152 (56%) | 16 (55%) | 136 (56%) |  |
| **Race, n (%)** |  |  |  | 0.12 |
| Others | 47 (17%) | 2 (6.9%) | 45 (19%) |  |
| White | 225 (83%) | 27 (93%) | 198 (81%) |  |
| **Marital status, n (%)** |  |  |  | 0.2 |
| Divorced | 102 (38%) | 14 (48%) | 88 (36%) |  |
| Married | 170 (63%) | 15 (52%) | 155 (64%) |  |
| **Household income, n (%)** |  |  |  | 0.5 |
| <60000 | 79 (29%) | 7 (24%) | 72 (30%) |  |
| 60000+ | 193 (71%) | 22 (76%) | 171 (70%) |  |
| **Rural urban, n (%)** |  |  |  | >0.9 |
| Metropolitan | 241 (89%) | 26 (90%) | 215 (88%) |  |
| Nonmetropolitan | 31 (11%) | 3 (10%) | 28 (12%) |  |
| **Tumor size, n (%)** |  |  |  | 0.3 |
| <4.5 | 139 (51%) | 12 (41%) | 127 (52%) |  |
| 4.5+ | 133 (49%) | 17 (59%) | 116 (48%) |  |
| **Primary site, n (%)** |  |  |  | 0.3 |
| Frontal lobe | 79 (29%) | 12 (41%) | 67 (28%) |  |
| Parietal lobe | 52 (19%) | 3 (10%) | 49 (20%) |  |
| Temporal lobe | 41 (15%) | 5 (17%) | 36 (15%) |  |
| Others | 100 (37%) | 9 (31%) | 91 (37%) |  |
| **Laterality, n (%)** |  |  |  | 0.7 |
| Left | 113 (42%) | 12 (41%) | 101 (42%) |  |
| Not a paired site | 53 (19%) | 4 (14%) | 49 (20%) |  |
| Right | 106 (39%) | 13 (45%) | 93 (38%) |  |
| **Surgery, n (%)** |  |  |  | <0.001 |
| NS | 81 (30%) | 8 (28%) | 73 (30%) |  |
| Biopsy | 56 (21%) | 2 (6.9%) | 54 (22%) |  |
| STR | 57 (21%) | 15 (52%) | 42 (17%) |  |
| GTR | 78 (29%) | 4 (14%) | 74 (30%) |  |
| **Radiotherapy, n (%)** | 179 (66%) | 19 (66%) | 160 (66%) | >0.9 |
| **Chemotherapy, n (%)** | 152 (56%) | 18 (62%) | 134 (55%) | 0.5 |
| ^1^n (%) ^2^Pearson's Chi-squared test; Fisher's exact test | | | | |
| NS, No surgery; STR,Subtotal resection; GTR, Gross total resection; SM, Synchronous metastasis | | | | |
