# Supplementary figures and images for: The prognostic significance of synchronous metastasis in glioblastoma multiforme patients: a propensity score-matched analysis using SEER data
Source: Front Neurol. 2024 Oct 8;15:1429826. doi: 10.3389/fneur.2024.1429826 (PMC11493671; doi:10.3389/fneur.2024.1429826)

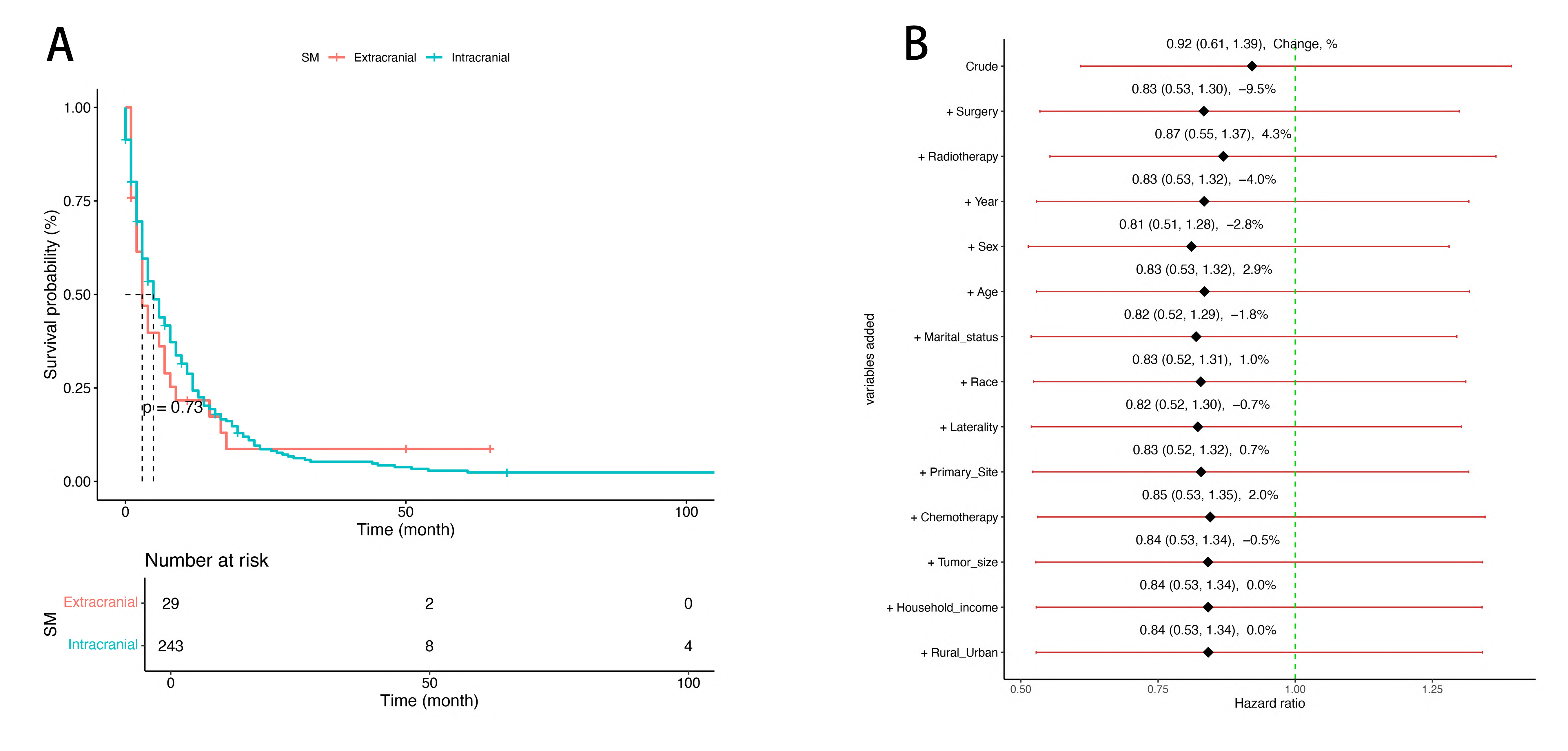

Supplement: Supplementary file 3 [file Image_1.JPEG]
